# Stillbirth Definitions in Transition: A Call for Updated, Nuanced Criteria

**DOI:** 10.7759/cureus.100681

**Published:** 2026-01-03

**Authors:** Sujata Siwatch, Gaurav Khastgir

**Affiliations:** 1 Obstetrics and Gynaecology, Post Graduate Institute of Medical Education and Research (PGIMER), Chandigarh, IND; 2 Obstetrics and Gynaecology, Employees State Insurance-Post Graduate Institute of Medical Sciences and Research (ESI-PGIMSR) - Employees State Insurance Corporation (ESIC) Medical College Hospital and Occupational Disease Centre, Kolkata, IND

**Keywords:** india, perinatal care, perinatal mortality, stillbirth, stillbirth prevention

## Abstract

Stillbirth remains a significant, yet under-recognized, global health challenge, complicated by wide variation in definitions and reporting practices. While advances in neonatal care have shifted viability thresholds from 28 to as low as 20 weeks in some countries, inconsistent criteria continue to hinder comparisons across settings. India contributes the largest number of stillbirths worldwide and, despite a steady decline over two decades, substantial disparities in perinatal care persist. Current Indian stillbirth reporting systems have significant drawbacks that limit the accuracy and comparability to global standards. Recent changes in legislation offer an opportunity to introduce a separate category for medical termination of pregnancy, improving accuracy. Strengthening stillbirth definitions, recognizing undelivered fetal deaths, and improving surveillance systems, such as through the Indian Council of Medical Research Stillbirth Pooled India Cohort (ICMR-SPIC) cohort, can generate clearer insights into risk factors and guide targeted interventions. Accurate classification is essential for reducing preventable stillbirths and advancing national and global goals.

## Editorial

The World Health Organization (WHO) defines stillbirth as a baby born without any signs of life at or after 28 weeks of gestation or with a birth weight of less than 1000 g [1]. It remains one of the most neglected tragedies worldwide, with 2.6 million stillbirths occurring globally each year [2]. Each death is a tragedy both for the parents and has an impact on the affected women, families, and healthcare providers. From a broader community and national perspective, it helps us identify fallacies in our perinatal care and health behaviors and provides an opportunity to address and improve them. The causes of stillbirth may be maternal, fetal, and placental. Finding and learning from them may make us wiser and help us avoid such tragedies, which often recur. This editorial focuses on identifying the drawbacks in current stillbirth reporting practices based on secondary data and global estimates.

The definition of stillbirth has evolved in the United States, shifting from 28 weeks to 26 weeks, then to 24 weeks, and now to 20 weeks [3]. These changes reflect progress in neonatal medical care and higher survival rates. However, comparing stillbirth rates across and within countries is challenging because of inconsistent definitions and incomplete data collection. The Every Newborn Action Plan (ENAP), endorsed by the World Health Assembly in 2014, aimed to lower the stillbirth rate (SBR) to below 12 per 1000 total births by 2030 [4]. India is committed to decreasing stillbirth and early neonatal mortality rates to under 10 per 1000 births through strategies outlined in the 2014 India Newborn Action Plan (INAP) [5]. While many high-income countries have met this goal, low- and middle-income countries in sub-Saharan Africa and South Asia still account for 98% of all stillbirths worldwide [6].

The Global Burden of Disease (GBD) study estimates that in 2021, India had 567,000 stillbirths at ≥20 weeks of gestation and 397,300 at ≥28 weeks, making it the highest worldwide [6]. As India accounts for the largest share of global stillbirths, improving stillbirth data collection in India has significant global implications for reducing this burden. Over two decades, India reduced SBR by 4% annually, with a 53% decline from 2000 to 2019 (from 29.6 to 13.9 stillbirths per 1000 births) [7]. Despite progress, the burden remains high, requiring improved maternal and perinatal healthcare to address ongoing issues and inequities. 

Similar to the WHO definition, Indian statistics also use 28 weeks as the cutoff for stillbirths. Disparities in the quality and availability of perinatal care across India are as varied as its customs, languages, and fabrics. While it may take time to standardize the cutoff for viability in stillbirth definitions, higher-level centers should adopt lower thresholds to improve the audit of their perinatal care quality.

India's national stillbirth reporting systems, the Health Management Information System (HMIS) and Sample Registration System (SRS), face methodological challenges that limit the accuracy and comparability of data. Significant underreporting occurs due to incomplete vital registration, inadequate recording of events in private, rural, tribal, and home settings, and social stigma around pregnancy loss. Moreover, misclassification between stillbirths and early neonatal deaths further distorts figures and complicates data validation. Additionally, substantial regional variation across states, driven by differences in healthcare infrastructure and reporting practices, creates disparities [8].

Following changes to the present Medical Termination of Pregnancy (MTP) laws in India, the gestational age bar for terminating pregnancies with major malformations has been lifted, as they carry a substantial risk of being incompatible with life [9]. If born, the child may have such severe physical or mental abnormalities that they become seriously handicapped. These cases are not counted as ‘stillbirths’ but rather as ‘congenital malformations’ in the Western world, like in the United Kingdom. However, in India, in the absence of such protocols, with even fatal malformations like aneuploidy, these children continue to be counted as ‘stillborn’. This would have further contributed to the higher stillbirth rates in India. Hence, for comparability of data in world statistics, the Government of India’s analysis of stillbirth rates should incorporate a separate category while counting babies born of medical termination of pregnancy, regardless of gestation or weight.

Another group of fetuses requiring attention are those who died undelivered with their mothers. Some cultures regard the child as a separate entity, deserving its own burial. Current classification systems, both in India and internationally, do not provide specific guidelines regarding this group. However, for medical statistics, these children should be classified separately based on their gestation for healthcare analysis and to evaluate the impact of pregnancy on morbidity and mortality across different health conditions.

Accurate and timely classification of stillbirths is crucial to reducing the SBR and meeting the ambitious targets of the ENAP and INAP, respectively. The Indian Council of Medical Research (ICMR) introduced the Stillbirth Pooled India Cohort Dataset (ICMR-SPIC) to study specific risk factors and their impact on SBR. Pooling data from various pregnancy cohorts provides robust insights into prevalence and region-specific risk factors. However, variability in data completeness across cohorts limited information on intrapartum care, behavioral risk factors, and the reliance on the last menstrual period for gestational age estimation constrains causal inference and necessitates cautious interpretation. Despite these limitations, ICMR-SPIC provides a unique platform for developing prediction models for high-risk pregnancies and informing evidence-based clinical guidelines, targeted interventions, and policy formulation [10].

Improving the quality of stillbirth data in India has the potential to strengthen healthcare delivery, reduce regional and socioeconomic disparities, guide evidence-based policy, and address the persistent social stigma surrounding stillbirth. Accurate data not only helps prevent avoidable stillbirths but also contributes to better maternal and neonatal health outcomes and progress toward global health goals. To shift from enumeration to prevention, the Ministry of Health and Family Welfare and the Government of India’s Technical Advisory Committee on Stillbirths must reform stillbirth reporting by harmonizing gestational age thresholds, mandating separate classification for the medical termination of pregnancy, and distinctly recording undelivered intrauterine fetal deaths. These reforms should be integrated into health systems, linked to perinatal audits, and aligned with ICD standards. Low- and middle-income countries like India face additional challenges in addressing preventable causes of stillbirth, including maternal infections, non-communicable diseases, anaemia, and malnutrition. It is therefore essential to comprehensively monitor the antenatal period, identify and manage high-risk cases, and ensure supervised institutional deliveries by trained birth attendants. Recognizing stillbirth as a loss of life, not just a baby born without life, is a crucial step toward the ambitious targets that lie ahead.
